# Vistusertib (dual m-TORC1/2 inhibitor) in combination with paclitaxel in patients with high-grade serous ovarian and squamous non-small-cell lung cancer

**DOI:** 10.1093/annonc/mdy245

**Published:** 2018-07-17

**Authors:** B Basu, M G Krebs, R Sundar, R H Wilson, J Spicer, R Jones, M Brada, D C Talbot, N Steele, A H Ingles Garces, W Brugger, E A Harrington, J Evans, E Hall, H Tovey, F M de Oliveira, S Carreira, K Swales, R Ruddle, F I Raynaud, B Purchase, J C Dawes, M Parmar, A J Turner, N Tunariu, S Banerjee, J S de Bono, U Banerji

**Affiliations:** 1 Department of Oncology, University of Cambridge and Cambridge University Hospitals NHS Foundation Trust, Cambridge; 2 Manchester Academic Health Science Centre, The University of Manchester and The Christie NHS Foundation Trust, Manchester; 3 Drug Development Unit, The Institute of Cancer Research and The Royal Marsden, London, UK; 4 Department of Haematology-Oncology, National University Health System, Singapore; 5 Centre for Cancer Research and Cell Biology, Queen’s University Belfast and Belfast City Hospital, Belfast; 6 School of Cancer and Pharmaceutical Sciences, King’s College London and Guy's and St Thomas’ NHS Foundation Trust, London; 7 Cardiff University and Velindre Cancer Centre, Cardiff; 8 University of Liverpool and Clatterbridge Cancer Centre NHS Foundation Trust, Wirral; 9 Department of Oncology, Oxford University Hospitals NHS Foundation Trust, Oxford; 10 University of Glasgow and Beatson West of Scotland Cancer Centre, Glasgow; 11 Oncology, IMED Biotech Unit AstraZeneca, Cambridge; 12 Clinical Trials and Statistics Unit, The Institute of Cancer Research, London; 13 Division of Clinical Studies, The Institute of Cancer Research, London; 14 Division of Cancer Therapeutics, The Institute of Cancer Research, London; 15 Department of Gynae-Oncology, The Royal Marsden, London, UK

**Keywords:** phase 1, m-TORC1/m-TORC2 inhibitor, combination therapy, ovarian cancer, squamous non-small-cell lung cancer

## Abstract

**Background:**

We have previously shown that raised p-S6K levels correlate with resistance to chemotherapy in ovarian cancer. We hypothesised that inhibiting p-S6K signalling with the dual m-TORC1/2 inhibitor in patients receiving weekly paclitaxel could improve outcomes in such patients.

**Patients and methods:**

In dose escalation, weekly paclitaxel (80 mg/m^2^) was given 6/7 weeks in combination with two intermittent schedules of vistusertib (dosing starting on the day of paclitaxel): schedule A, vistusertib dosed bd for 3 consecutive days per week (3/7 days) and schedule B, vistusertib dosed bd for 2 consecutive days per week (2/7 days). After establishing a recommended phase II dose (RP2D), expansion cohorts in high-grade serous ovarian cancer (HGSOC) and squamous non-small-cell lung cancer (sqNSCLC) were explored in 25 and 40 patients, respectively.

**Results:**

The dose-escalation arms comprised 22 patients with advanced solid tumours. The dose-limiting toxicities were fatigue and mucositis in schedule A and rash in schedule B. On the basis of toxicity and pharmacokinetic (PK) and pharmacodynamic (PD) evaluations, the RP2D was established as 80 mg/m^2^ paclitaxel with 50 mg vistusertib bd 3/7 days for 6/7 weeks. In the HGSOC expansion, RECIST and GCIG CA125 response rates were 13/25 (52%) and 16/25 (64%), respectively, with median progression-free survival (mPFS) of 5.8 months (95% CI: 3.28–18.54). The RP2D was not well tolerated in the SqNSCLC expansion, but toxicities were manageable after the daily vistusertib dose was reduced to 25 mg bd for the following 23 patients. The RECIST response rate in this group was 8/23 (35%), and the mPFS was 5.8 months (95% CI: 2.76–21.25).

**Discussion:**

In this phase I trial, we report a highly active and well-tolerated combination of vistusertib, administered as an intermittent schedule with weekly paclitaxel, in patients with HGSOC and SqNSCLC.

**Clinical trial registration:**

ClinicialTrials.gov identifier: CNCT02193633

**Key Message**

This phase IB study established a dose and schedule of vistusertib (dual m-TORC1/2 inhibitor) in combination with weekly paclitaxel. Response rates in pre-treated populations of patients in the expansion cohorts of HGSOC and squamous NSCLC were 52% and 35%, respectively. Progression-free survival in both cohorts was 5.8 months. Randomised trials of this combination are warranted.

## Introduction

We have previously studied cancer cells isolated from serous effusions and shown raised p-S6K to be associated with chemoresistance and poor clinical outcome in ovarian and lung cancers, respectively [1, 2]. This led us to hypothesise that inhibition of m-TOR signalling, in combination with chemotherapy, could improve treatment outcomes in these tumour types.

Analogues of rapamycin such as everolimus have been recognised to inhibit only m-TORC1 and not m-TORC2 in the m-TOR complex [3]. The dual m-TORC1/2 inhibitor vistusertib (AZD2014) has a short half-life, giving greater flexibility for intermittent dosing schedules [4, 5]. Weekly paclitaxel was chosen as the chemotherapy backbone, as it is often used to treat advanced ovarian cancer. Pre-clinical studies of vistusertib and paclitaxel revealed an additive effect on growth *in vitro* and *in vivo*, with the combination showing increased apoptosis and metabolic effects consistent with the mechanism of action of vistusertib [6].

Here, we report the results of the TAX-TORC study, a phase IB dose-escalation study, with a pre-planned dose-expansion cohort in HGSOC and an additional expansion cohort in sqNSCLC (supplementary Figure S1, available at *Annals of Oncology* online).

## Patients and methods

### Conduct of the study

The academic sponsors of this study were The Institute of Cancer Research and The Royal Marsden (CCR3667), and the trial was reviewed by a central research ethics committee (REC ref: 13/LO/0066). The study was funded by AstraZeneca. Nine Experimental Cancer Medicine Centres across the UK participated in this study. All patients were treated after obtaining written, informed consent. Cancer Research UK trial number: CRUKD/12/013.

*Inclusion/exclusion criteria:* Inclusion criteria in the dose-escalation arm included an ECOG performance status of 0 or 1. Haematological and biochemistry criteria were standard for phase I studies, and details are available in the supplementary data, available at *Annals of Oncology* online.

### Treatment

The dose of paclitaxel administered was 80 mg/m^2^ once weekly for 6/7 weeks in a 7-week cycle. In the first week of the dose-escalation cohorts, patients received only paclitaxel on C1D1, then vistusertib on C1D3 to allow for PK and PD samplings. Patients then received weekly paclitaxel (on days 8, 15, 22, 29, and 36) with vistusertib, also starting on days 8, 15, 22, 29, and 36, given orally twice daily either for three consecutive days per week (schedule A: 3/7 days, 6/7 weeks) or two consecutive days per week (schedule B: 2/7 days, 6/7 weeks). In the dose expansion, schedule A was taken forward with patients dosing with vistusertib weekly on days 1–3 for 6 weeks of a 7-week cycle.

### Evaluation of toxicity

NCI-CTCAE V4·0 was used to assess toxicity.

### Evaluation of response

RECIST v1.1 was used to assess tumour response supported by GCIG CA125 response in patients with HGSOC. Response was assessed at the end of every 7-week cycle.

### Pharmacokinetic and pharmacodynamic evaluations

Pharmacokinetic (PK) sampling was carried out for all patients in the dose-escalation arm for 24 h on C1D1 (paclitaxel alone), C1D3 (vistusertib alone), and on C1D1 (combination of paclitaxel and vistusertib). PD sampling was carried out for all patients in the dose-escalation arm. Sampling for PD assays was carried out on the same days as PK sampling. Phosphorylation of AKTSer473 (Ser^473^ p-AKT) was quantified in platelet-rich plasma (PRP) (for detailed methods, see supplementary data, available at *Annals of Oncology* online) [7].

### Sequencing

DNA was extracted from formalin-fixed and paraffin-embedded (FFPE) tumour blocks. In addition, circulating free DNA (cfDNA) when collected at baseline, at the end of cycle 1 and, where possible, at progression, was extracted from 4 to 8 mL of plasma. Sequencing libraries were constructed using a customised Generead DNAseq Mix-n-Match v2 panel (Qiagen) covering 4841 amplicons (310, 077 bp) across 67 genes. Libraries were run using the MiSeq Sequencer (Illumina); sequence alignment and mutation calling were performed.

### Methods—statistical analysis

The data cut-off for this article was 1 October 2017. Demographics were analysed by descriptive statistics. Safety was assessed in all enrolled patients. Patients considered not assessable for response had no post-baseline CT scan. The number of patients required for the dose-escalation phase was dependent on toxicities observed as the trial progressed. No formal power calculations were done.

Progression-free survival was estimated by the Kaplan–Meier method, beginning on the day of the first dose (C1D1) and continuing until disease progression. Patients who came off study for reasons other than disease progression (clinical or RECIST) were censored. This study is registered with ClinicalTrials.gov, identifier: NCT02193633.

## Results

### Dose-escalation cohort

#### Toxicity

Twenty-two patients were recruited to the dose-escalation cohort. The most common tumours were ovarian and lung cancers (supplementary Table S1, available at *Annals of Oncology* online). In the dose-escalation phase, vistusertib was tested at 25 mg, 50 mg, and 75 mg bd 3/7 days, 6/7 weeks (schedule A) with no dose limiting toxicities (DLTs) in the 25 mg or 50 mg groups. Two of the three patients in the 75 mg group experienced DLTs of fatigue and mucositis. Vistusertib was then tested at 50 mg and 75 mg bd 2/7 days (schedule B) with no DLTs. However, two of the three patients taking 100 mg bd 2/7 days experienced DLTs of rash (supplementary Figure S2, available at *Annals of Oncology* online). The maximally tolerated dose (MTD) of schedule A was, thus, 80 mg/m^2^ weekly paclitaxel with 50 mg vistusertib bd 3/7 days for 6/7 weeks in a 7-week cycle, with dosing starting concurrently on day 1 of each week. The MTD of schedule B was 80 mg/m^2^ weekly paclitaxel with 75 mg vistusertib bd 2/7 days for 6/7 weeks in a 7-week cycle, with dosing starting concurrently on day 1 of each week. The most common toxicities across both schedules were predominantly grade 1–2 fatigue, nausea, anaemia, and diarrhoea (Table 1), which are similar to that seen with weekly paclitaxel administration.

**Table 1.** Toxicity in the dose-escalation arm

Adverse event	Escalation 3d on, 4d off						Escalation 2d on, 5d off				Total (*N* = 21)
	25 mg (*N* = 3)		50 mg (*N* = 6)		75 mg (*N* = 3)		75 mg (*N* = 6)		100 mg (*N* = 3)		
	Grades 1–2	Grades 3–4	Grades 1–2	Grades 3–4	Grades 1–2	Grades 3–4	Grades 1–2	Grades 3–4	Grades 1–2	Grades 3–4	
Fatigue	3	0	5	0	0	3	1	2	1	1	16
Nausea	3	0	4	0	0	0	4	0	2	0	13
Anaemia	2	0	4	0	2	0	2	0	2	0	12
Diarrhoea	1	0	3	1	1	1	3	0	1	0	11
Peripheral sensory neuropathy	1	0	2	0	1	0	3	0	2	0	9
Skin rash	1	0	1	0	1	0	3	0	1	2	9
Alopecia	1	0	4	0	2	0	1	0	0	0	8
Dysgeusia	0	0	3	0	1	0	4	0	0	0	8
Mucositis	1	0	2	0	1	1	1	0	1	0	7
Neutropenia	0	0	2	1	1	2	0	0	1	0	7
Dyspepsia/gastric reflux	1	0	1	0	0	0	1	0	2	0	5
Hypophosphataemia	1	0	1	0	0	3	0	0	0	0	5
Pain	0	0	1	0	1	0	1	0	1	0	4
Paronychia	0	0	3	0	0	0	1	0	1	0	5

All drug-related events (possibly, probably and, definitely related) seen in more than 20% of patients in the dose-escalation cohorts. A total of 22 patients were treated in the dose escalation. One patient was treated with vistusertib on schedule B at 50 mg instead of 100 mg owing to urgent reporting of two dose-limiting toxicities. The patient did not have grade 3 or 4 toxicity or a dose-limiting toxicity, was evaluable, but has not been represented in the table for simplicity.

#### Pharmacokinetics

In all schedules tested, the PK of paclitaxel when administered alone or in combination with vistusertib was similar (Table 2). The PK of vistusertib alone or vistusertib in combination with paclitaxel in both schedules was comparable with previous single agent studies [5] (data not shown). The areas under the curve (AUC) *versus* dose of vistusertib was approximately dose proportional (supplementary Figure S3, available at *Annals of Oncology* online). Altogether, these suggest that there is no drug-drug PK interaction on drug exposure for either paclitaxel or vistusertib in combination compared with either agent administered alone.

**Table 2.** Pharmacokinetic profile of vistusertib

Variable	Day	AZD2014/paclitaxel							
		25 mg/80 mg		50 mg/80 mg		75 mg/80 mg		100 mg/80 mg	
		Geometric mean	*N*	Geometric mean	*N*	Geometric mean	*N*	Geometric mean	*N*
AUClast (h*ng/mL)	3	2090 (1290–3462)	3	2602 (708–11486)	7	7543 (4192–16542)	9	7556 (4188–12884)	3
	8	1054 (181–2785)	3	2026 (800–6137)	7	5209 (1576–13363)	8	7347 (4875–13997)	3
Cmax (ng/mL)	3	579 (478–785)	3	840 (462–3580)	7	1840 (983–2870)	9	1960 (1180–2670)	3
	8	248 (80–507)	3	500 (244–764)	7	1122 (442–1920)	8	1830 (1490–2420)	3
HL Lambda_z (h)	3	3.3 (2.3–4.2)	3	1.8 (0.8–3.2)	6	2.7 (1.2–5.9)	8	3.0 (2.5–3.3)	3
	8	3.5 (1.9–6.1)	3	2.2 (1.7–2.9)	6	3.1 (1.7–9.7)	8	2.8 (1.2–5.3)	3

The area under the curve (AUC), maximal concentration (Cmax), and half-life (HL) of vistusertib on C1D3 (administered as a single agent) and C1D8 (administered in combination with paclitaxel) across the different dose levels in the dose-escalation cohort.

#### Pharmacodynamics

**Figure 1.**
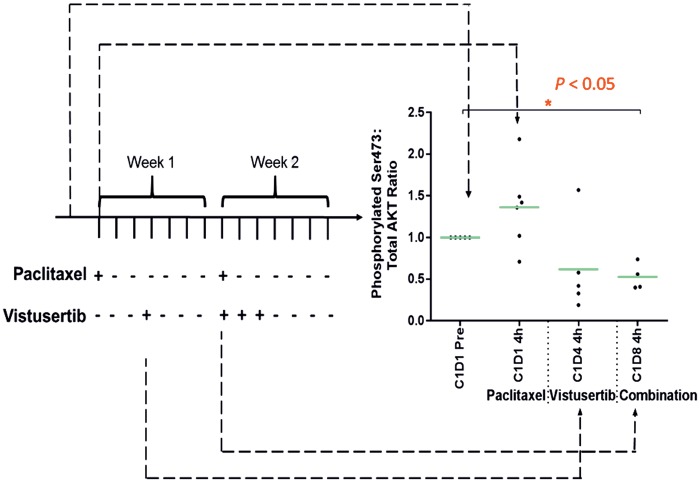
Pharmacodynamic profile of vistusertib at 50 mg bd 3/7. Phosphorylation of AKT (Ser473) in platelet-rich plasma was quantified using MSD electrochemiluminescent immunoassays and normalised to corresponding total AKT values. Baseline values were established prior to the start of treatment. On C1D1, only paclitaxel (80 mg/m^2^) was administered and a non-significant rise in p-ATK at 4 h following treatment was noted. On C1D4, a single dose of vistusertib was administered and non-significant reduction of p-AKT was seen. On C1D8, the combination of paclitaxel and vistusertib was administered, which caused a significant reduction of p-AKT compared with baseline. Points represent individual patients, orange line represents mean of up to *N* = 6 patients. Four samples were excluded because of haemolysis, which interfered with the assay (*P<0.05; paired *t*-test).

At the recommended phase II dose (RP2D) level of 50 mg bd of vistusertib and 80 mg/m^2^ of paclitaxel, there was a statistically non-significant increase in levels of Ser^473^ p-AKT at 4 h following 80 mg/m^2^ paclitaxel (1.4 fold; *P *=* *0.14). Vistusertib (50 mg bd 3/7) in addition to paclitaxel produced a reduction in Ser^473^ p-AKT at 4 h post-vistusertib to 53% of pre-dose levels (*P *=* *0.0495). This was 62% lower than the corresponding time-point following paclitaxel alone, suggesting that, at the RP2D of the combination, there is a significant reduction in p-AKT levels in normal tissue compared with baseline (Figure 1).

#### RP2D

In combination with weekly paclitaxel administered at 80 mg/m^2^ once weekly, the MTD of vistusertib was 50 mg bd (3/7 days) (schedule A) or 75 mg bd (2/7 days) (schedule B). Both doses had acceptable PK and PD profiles and would be acceptable as per the pharmacological audit trail [8]. Weekly vistusertib 50 mg bd 3 days on/4 days off combined with weekly paclitaxel 80 mg/m^2^ was taken forward as the RP2D based on reduced occurrence of grade 3 fatigue in this cohort.

#### Ovarian cancer expansion

**Figure 2.**
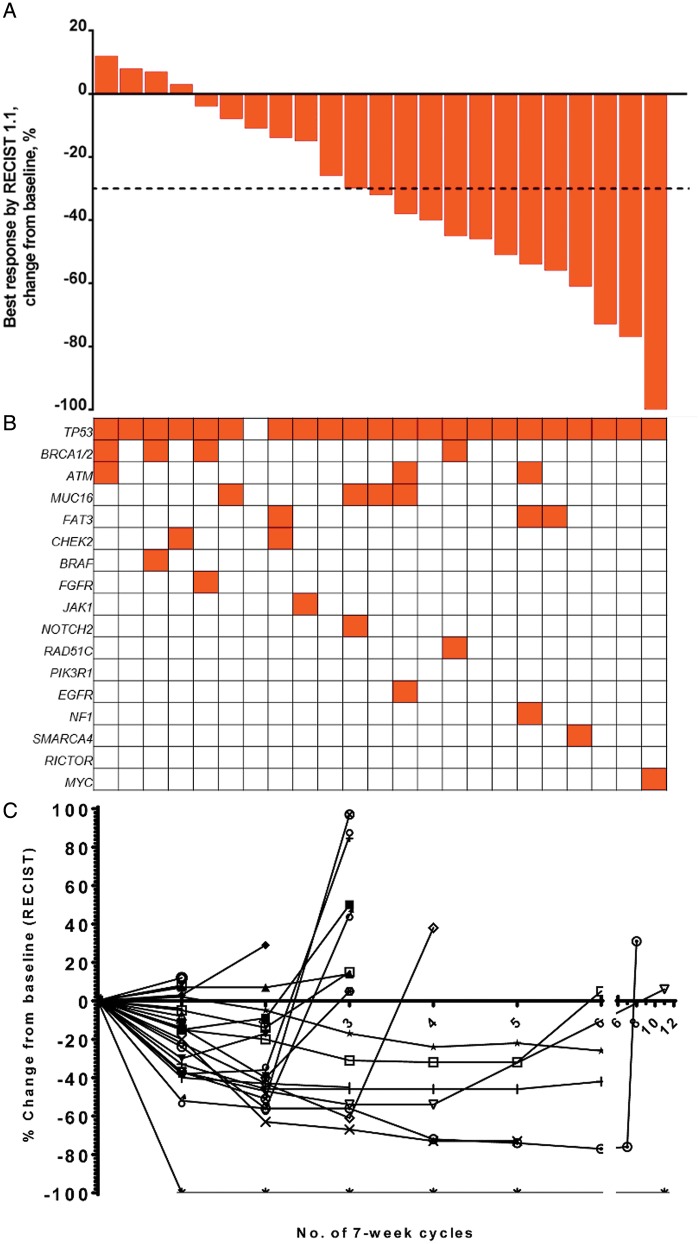
Clinical outcomes of patients in the ovarian cancer expansion treated at the R2PD for ovarian cancer. (A) Waterfall plot of 23/25 patients with ovarian cancer treated at the RP2D for ovarian cancer that were evaluable for response; two patients clinically progressed with bowel obstruction in the first cycle and did not have a repeat CT scan to assess response. A total of 19 of 25 (76%) patients showed a reduction in size of their tumour, with 13/25 (52%) achieving a partial response. (B) Mutations in tumour tissue or plasma of patients compared with clinical response. (C) Spider plots representing percentage change in measured sum of tumour dimensions of individual patients over time (each cycle is 7 weeks).

Twenty-seven patients with relapsed/refractory HGSOC were treated at the RP2D. Two patients were replaced as per protocol and were not considered for assessment of response. The median number of previous treatments was three: the majority (26/27; 96%) of patients having received paclitaxel and 3/27 patients (11%) having previously received weekly paclitaxel (supplementary Table S2, available at *Annals of Oncology* online). The RECIST and CA125 response rates were 13/25 (52%) and 16/25 (64%), respectively (Figure 2A). The mPFS was 5·8 months (95% CI: 3.3–18.5).

DNA sequencing (targeted panel of 67 genes) of FFPE tissue revealed that the most common mutation was TP53 detected in 23/25 (92%) patients. There was no correlation between specific mutations and response (Figure 2B).

#### Squamous lung cancer expansion

**Figure 3.**
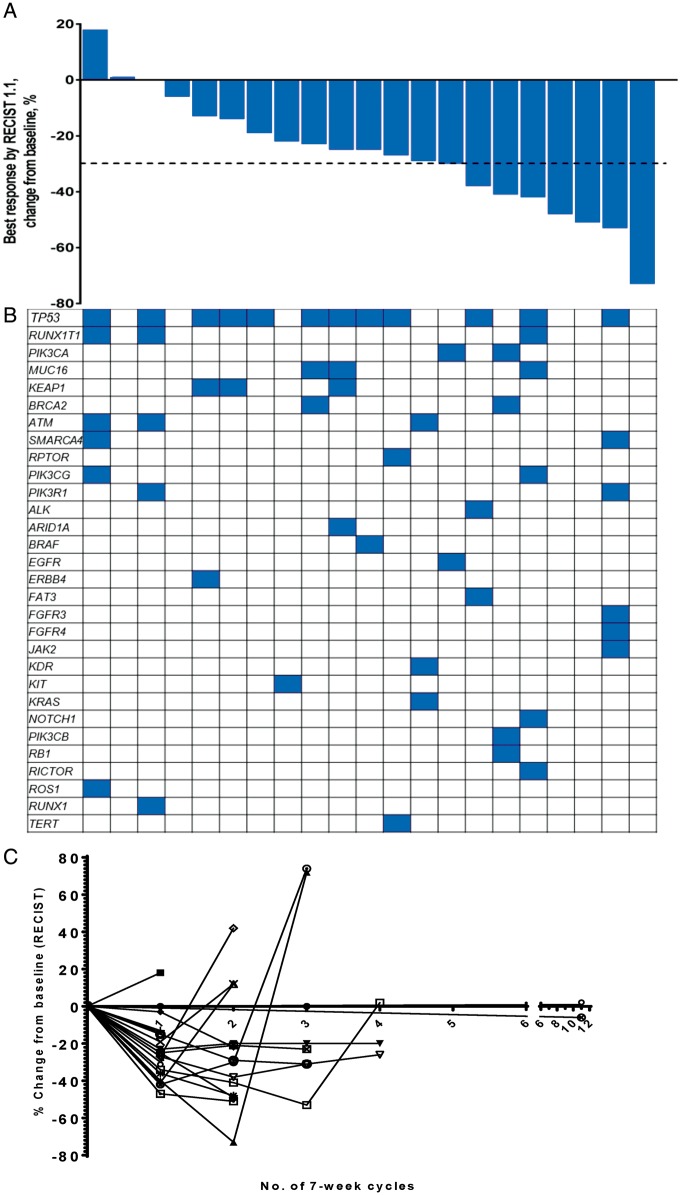
Clinical outcomes of patients in the squamous NSCLC expansion treated at the R2PD for squamous NSCLC. (A) Waterfall plot of 21/23 patients with sqNSCLC treated at RP2D of the combination; two patients clinically progressed within their first cycle and repeat radiological evaluation was not done. Eighteen of the 23 (78%) patients showed reduction in the size of their tumour with 8/23 (35%) achieving a partial response. (B) Mutations in tumour tissue or plasma of patients compared with clinical response. (C) Spider plots representing percentage change in measured sum of tumour dimensions of individual patients over the time (each cycle is 7 weeks).

Following two partial responses in patients with sqNSCLC in the dose-escalation cohort, we conducted a dose expansion in a cohort of 40 patients, starting at the RP2D of 80 mg/m^2^ paclitaxel and 50 mg vistusertib bd 3/7 days. This schedule was poorly tolerated, with fatigue, diarrhoea, and pneumonia being seen more frequently than in the dose-escalation cohort (supplementary Table S3A, available at *Annals of Oncology* online). The safety review committee reviewed the data of the first 17 patients and decided to reduce the dose of vistusertib to 25 mg bd 3/7 days for the remaining 23 patients due to be treated in this cohort. This dose was known to be pharmacodynamically active [5] and was better tolerated (supplementary Table S3B, available at *Annals of Oncology* online). The RECIST response rate in patients with sqNSCLC in the 25 mg cohort was 8/23 (35%) (Figure 3A), with an mPFS of 5.8 months (95% CI 2.8–21.3). Two patients with *PIK3CA* mutations showed partial responses, but there were no clear patterns linking mutations to response (Figure 3B).

## Discussion

We report the first study of the combination of weekly paclitaxel with the dual m-TORC1/2 inhibitor, vistusertib, establishing a safe dose and schedule and preliminary evidence of efficacy in HGSOC and SqNSCLC. We chose to investigate the m-TORC1/2 inhibitor in the context of weekly paclitaxel, as this regimen is often used in the setting of platinum-resistant ovarian cancer [9], and taxanes are commonly used in the treatment of platinum-resistant NSCLC (with comparable efficacy between weekly paclitaxel and docetaxel and better tolerability profile) [10, 11].

Toxicities of fatigue, nausea, anaemia, and diarrhoea in this dose-escalation cohort were not dissimilar to previous studies combining m-TOR inhibitors such as everolimus [12], ridaforolimus [13], or the m-TORC1/2 inhibitor, MLN028 [14], with weekly paclitaxel regimens. Hyperglycaemia, which has been commonly reported with m-TOR inhibitors, occurred at a very low incidence in our study [all grades: *N *=* *8 (11%), grade 3/4: *N = *1(1%)]. It was noted that many earlier studies were in breast cancer, where weekly paclitaxel is often used as standard-of-care. Of interest, in our study, patients with heavily pre-treated HGSOC tolerated vistusertib at 50 mg bd 3 days per week in combination with weekly paclitaxel. However, patients with sqNSCLC needed a dose reduction of vistusertib to 25 mg bd 3 days per week. Patients with sqNSCLC often exhibit risk factors and co-morbidities that correlate with poor tolerance of chemotherapy such as hypoxia, a history of smoking, and pulmonary fibrosis [15]. We have previously reported on the increased risk of infections of patients treated with PI3K pathway inhibitors used as part of combination therapy [16]. In our experience, this is the first time that it has been necessary to recommend two separate doses for different tumour types within the same study.

The PK profile of vistusertib was not significantly different from previous reports in single-agent studies [5] and was no different when administered alone or in combination with paclitaxel. The pharmacodynamic profile of vistusertib in PRP showed administration of vistusertib led to abrogation of AKTSer473 phosphorylation, providing proof-of-principle of the desired biological effect of inhibiting the PI3K–Akt-m-TOR pathway.

The clinical outcomes of patients receiving the combination of weekly paclitaxel and vistusertib in this non-randomised phase I expansion were encouraging for the patient groups explored. In the ovarian cohort, the three median lines of previous treatment were: 12% of patients were platinum-refractory, 48% had progressed within 6 months of the last platinum treatment, and 96% had progressed within a year of their last platinum treatment. In this cohort, the RECIST and CA125 response rates were 52% and 64%, respectively, with a progression-free interval of 5.8 months, which is better than historic data reported for the use of weekly paclitaxel therapy [9]. The control chemotherapy arm of a contemporary phase III study studying the addition of bevacizumab to chemotherapy in the setting of 2^nd^ or 3^rd^ line chemotherapy in a platinum-resistant disease state achieved a response rate of 12% and progression-free survival was 3.9 months [17]. The results of the TAX-TORC study have led to a randomised phase II study of weekly paclitaxel *versus* paclitaxel and vistusertib, which is ongoing (ISRCTN16426935) [18].

The standard-of-care of sqNSCLC changed with the introduction of immune checkpoint inhibitors with response rates of ∼15% in patient cohorts not selected for PD-L1 expression confirmed by randomised control trials [19]. In the TAX-TORC study, at the tolerated doses of paclitaxel (80 mg/m^2^/week) and vistusertib (25 mg bd 3/7 days), the response rate and progression-free survival was 35% and 5.8 months, respectively. These data exceed traditional outcomes for the sqNSCLC population beyond first-line therapy and demonstrate potential for benefit and warrant further evaluation. A possible use of this regimen could be in the setting of patients with sqNSCLC who do not have expression of PD-L1 [20].

We attempted to identify biomarkers of response to the combination by studying a panel of 67 genes that were known to be commonly mutated in HGSOC [21] and sqNSCLC [22]. The mutations found in our study were in keeping with those described elsewhere in these tumour types; however, there were no significant differences in mutation profiles of responders and non-responders in this small dataset.

### Conclusion

We report a phase I study combining weekly paclitaxel and a dual m-TORC1/2 inhibitor, vistusertib, with expansions in HGSOC and squamous NSCLC, which are both areas of unmet need. The trial showed tolerable schedules in expansion cohorts of over 20 patients. The response rates and progression-free survival in these non-randomised phase I expansions show promise, and randomised phase II studies are recommended to study these combinations further.

## Supplementary Material

Supplementary Fig1 S1

Supplementary Fig2 S2

Supplementary Fig3 S3

Supplementary Data

Supplementary Table 1

Supplementary Table 2

Supplementary Table 3A 3B

## Notes

Note: This study was previously presented as a poster at the ASCO Annual Meeting, June 2017 [abstr 2571]; as an oral presentation at the TAT Congress, March 2017 [abstr 03.2]; as a poster at the 17^th^ World Conference on Lung Cancer, Dec 2016 [abstr P3.02C-003]; as a poster discussion at the ESMO Meeting, October 2016 [abstr 362PD]; as an oral Plenary presentation at the AACR Annual Meeting, April 2015 [abstr CT138]; as an oral presentation at SEOM, October 2014; as a poster at the ESMO Meeting, September 2014 [abstr 468P]; as a poster at the ASCO Annual Meeting, June 2014 [abstr 2607]; and as an oral presentation at the Targeted Anticancer Therapies Congress, March 2014 [abstr O3.2]. Pharmacodynamic data of five patients (not all patients) with HGSOC have been used in a previous publication [Wong Te Fong AC et al, *Oncotarget* 2017; **8**(69): 113874-84].
