# Supplementary figures and images for: Vistusertib (dual m-TORC1/2 inhibitor) in combination with paclitaxel in patients with high-grade serous ovarian and squamous non-small-cell lung cancer
Source: Ann Oncol. 2018 Jul 17;29(9):1918–25. doi: 10.1093/annonc/mdy245 (PMC6158767; doi:10.1093/annonc/mdy245)

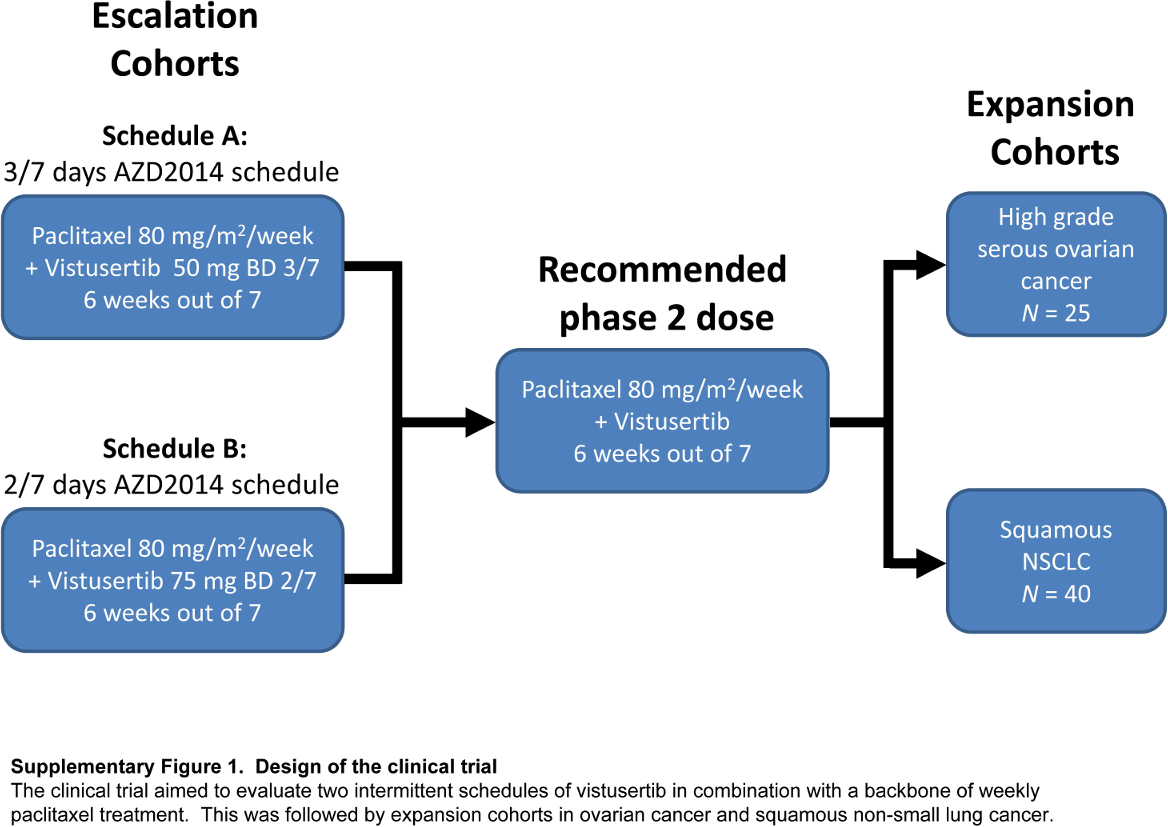

Supplement: Supplementary Fig1 S1 [file mdy245_fig1_s1.png]

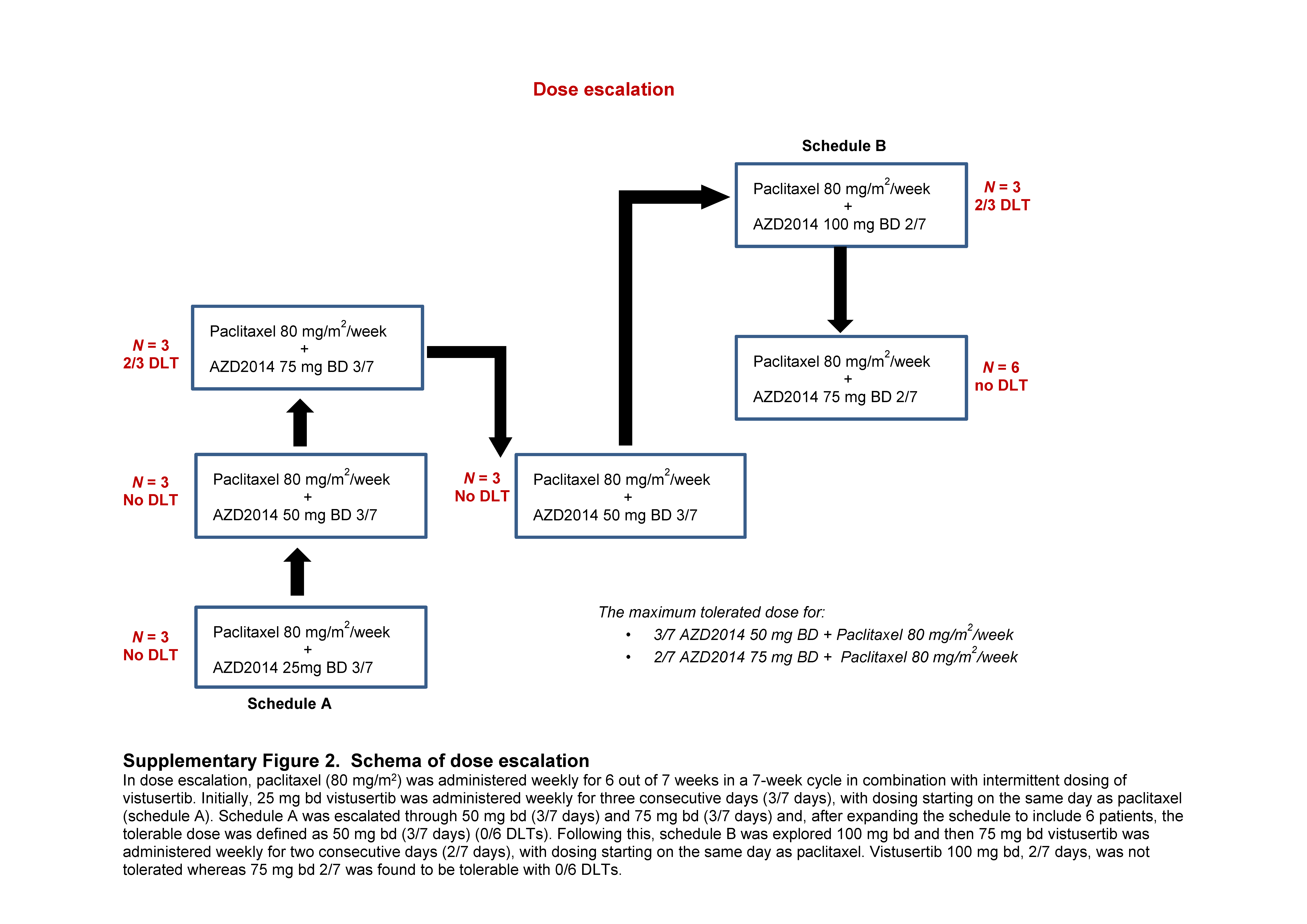

Supplement: Supplementary Fig2 S2 [file mdy245_fig2_s2.png]

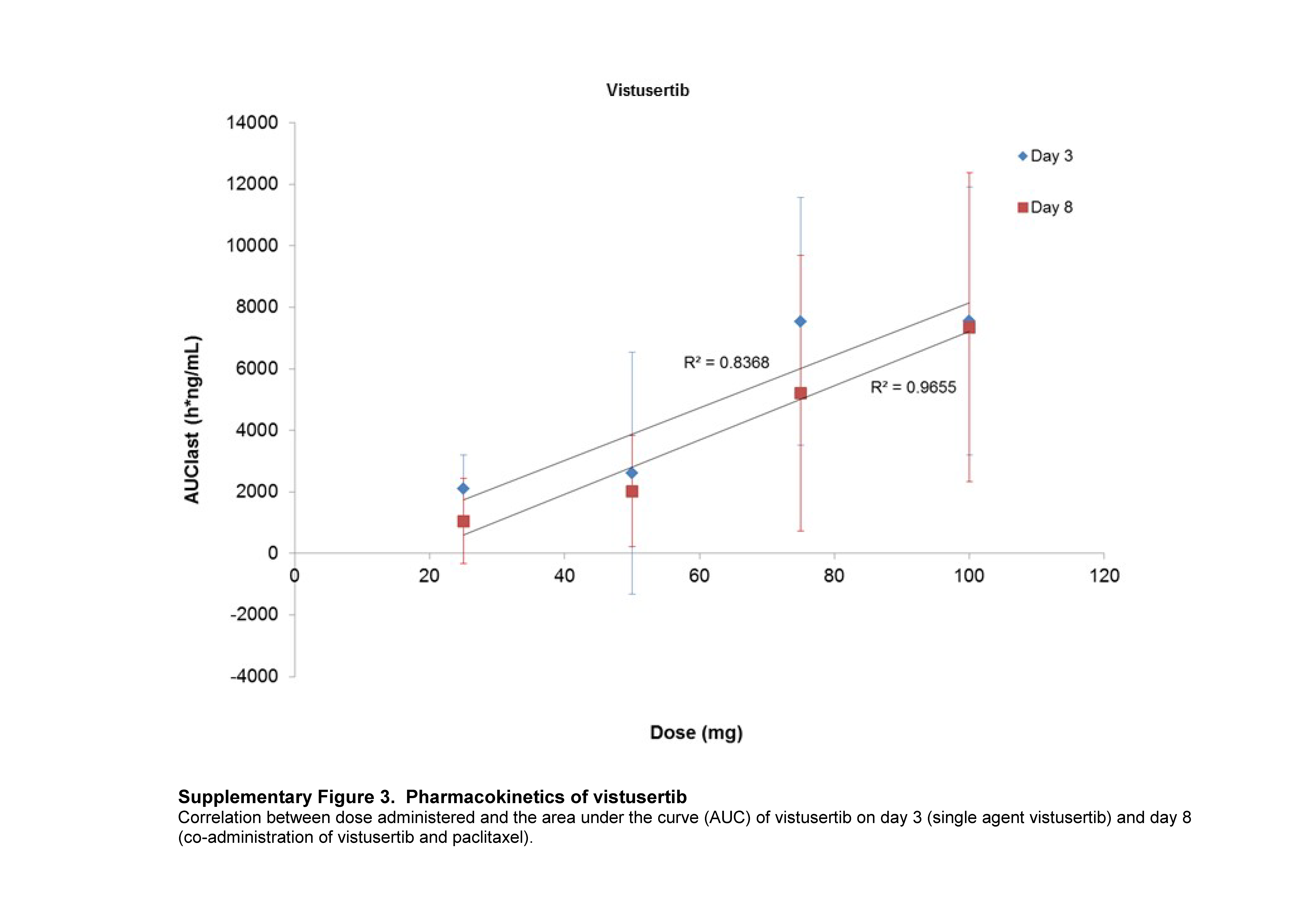

Supplement: Supplementary Fig3 S3 [file mdy245_fig3_s3.png]
