# Supplementary material for: Vistusertib (dual m-TORC1/2 inhibitor) in combination with paclitaxel in patients with high-grade serous ovarian and squamous non-small-cell lung cancer
Source: Ann Oncol. 2018 Jul 17;29(9):1918–25. doi: 10.1093/annonc/mdy245 (PMC6158767; doi:10.1093/annonc/mdy245)
Supplement: Supplementary Data [file mdy245_supplementary_data_revised.docx]

**Supplementary data**

**Inclusion and exclusion criteria**

**Inclusion Criteria:**

|  | Histologically- or cytologically-proven solid tumour refractory to conventional treatment, or for which no conventional therapy exists or is declined by the patient, or where treatment with paclitaxel is an appropriate treatment option. Patients will be enrolled into two cohorts on the expansion phase. One cohort of the patients must have recurrent high grade serous ovarian cancer. Patients with clear cell ovarian are excluded. The other cohort of patients at expansion must have squamous cell lung cancer. | |
| --- | --- | --- |
|  | Patients who have had conventional treatment and where paclitaxel is appropriate. In instances where paclitaxel is appropriate but the patient has not already received it the patient may be enrolled after discussion between the referring oncologist and Principal Investigator. | |
|  | Life expectancy of at least 12 weeks | |
|  | ECOG performance status of 0-1 (Appendix 1) | |
|  | Females should be using adequate contraceptive measures (see restrictions below), should not be breast-feeding and must have a negative pregnancy test prior to start of dosing if of child-bearing potential or must have evidence of non-child-bearing potential by fulfilling one of the following criteria at screening:  Post-menopausal defined as aged more than 50 years and amenorrhoeic for at least 12 months following cessation of all exogenous hormonal treatments  Documentation of irreversible surgical sterilisation by hysterectomy, bilateral oophorectomy or bilateral salpingectomy but not tubal ligation | |
|  | Male patients should be willing to use barrier contraception i.e., condoms | |
|  | Measurable or evaluable disease. Patients enrolled in the expansion phase should have measurable disease by RECIST v1.1 criteria (Appendix 3). | |
|  | Haematological and biochemical indices within the ranges shown below. These measurements must be performed within one week (Day -7 to Day 1) before the patient goes in the trial. | |
|  | **Laboratory Test** | **Value required** |
|  | Haemoglobin (Hb) | ≥ 9.0 g/dL |
|  | Absolute neutrophil count | ≥ 1.5 x 10^9^/L |
|  | Platelet count | ≥ 100 x 10^9^/L |
|  | Serum bilirubin | ≤ 1.5 x upper limit of normal (ULN) |
|  | Alanine aminotransferase (ALT) or aspartate aminotransferase (AST) | ≤ 2.5 x (ULN) if no demonstrable liver metastases or ≤ 5 times ULN in the presence of liver metastases |
|  | Alkaline phosphatase (ALP) | < 5 x ULN |
|  | Creatinine clearance  OR  Serum creatinine | ≥ 50 mL/min (uncorrected value)  ≤ 1.5 x ULN |
|  | Fasting glucose | ≤ 125 mg/dL (7 mmol/L) |
|  | Erythrocyte-HbA1c | ≤ 59 mmol/mol |

1. 18 years or over
2. Written (signed and dated) informed consent and be capable of co-operating with treatment and follow-up

**Exclusion criteria:**

|  | Radiotherapy (except for palliative reasons), chemotherapy, endocrine therapy, or immunotherapy during the previous 3 weeks (4 weeks for investigational medicinal products and 6 weeks for nitrosoureas and Mitomycin-C) before treatment.  N.B. Exceptions to this are patients receiving weekly Taxol as standard of care who have not had a partial or complete response after 6 to 12 weekly doses. Those patients should discontinue their weekly Taxol treatment and may be enrolled to the dose expansion phase without a wash out period. |
| --- | --- |
|  | Ongoing toxic manifestations of previous treatments. Exceptions to this are alopecia or certain Grade 1 toxicities, which in the opinion of the Investigator and the DDU should not exclude the patient. |
|  | Known leptomeningeal involvement, brain metastases or spinal cord compression |
|  | Known hypersensitivity (>Grade 2) to taxanes, drugs containing Cremophor, AZD2014 or structurally/chemically similar drugs |
|  | Unresolved bowel obstruction |
|  | Current refractory nausea and vomiting, chronic gastrointestinal disease, inability to swallow formulated product or previous significant bowel resection that would preclude adequate absorption of AZD2014 |
|  | Patients with Diabetes Type I or uncontrolled Type II (HbA1c >59 mmol/mol assessed locally) as judged by the investigator |
|  | Major surgery within 4 weeks prior to entry to the study (excluding placement of  vascular access), or minor surgery within 2 weeks of entry into the study and from which the patient has not yet recovered |
|  | Treatment with warfarin. Patients on warfarin for DVT/PE can be converted to LMWH. |
|  | Exposure to potent or moderate inhibitors or inducers of CYP3A4/5 if taken within the stated washout periods before the first dose of study treatment:   - Inhibitors (competitive): ketoconazole, itraconazole, indinavir, saquinovir, nelfinavir, atazanavir, amprenavir, fosamprenavir, troleandomycin, telithromycin, fluconazole, nefazodone, cimetidine, aprepitant, miconazole, fluvoxamine, P-glycoprotein, grapefruit juice, or Seville oragnes (1 week minimum wash-out period), amiodarone (27 week minimum wash-out period) - Inhibitors (time dependent): erythromycin, clarithromycin, verapamil, ritonavir, diltiazem (2 week minimum wash-out period) - Inducers: phenytoin, rifampicin, St. John's Wort, carbamazepine, primidone, griseofulvin, carbamazepine, barbiturates, troglitazone, pioglitazone, oxcarbazepine, nevirapine, efavirenz, rifabutin (3 week minimum wash-out period) and phenobarbitone (5 week minimum washout period) |
|  | Exposure to potent or moderate inhibitors or inducers of CYP2C8 if taken within the stated washout periods before the first dose of study treatment:   - Inhibitors: Gemfibrozil, trimethoprim, glitazones, montelukast, quercetin (1 week minimum wash-out period) - Inducers: Rifampicin (3 week minimum wash-out period) |
|  | At high medical risk because of non-malignant systemic disease including active uncontrolled infection e.g. interstitial lung disease, severe hepatic impairment, uncontrolled chronic renal disease |
|  | Known to be serologically positive for hepatitis B, hepatitis C or human immunodeficiency virus (HIV). |
|  | Patients who have experienced any of the following procedures or conditions  currently or in the preceding 12 months:   - coronary artery bypass graft - angioplasty - vascular stent - myocardial infarction (MI) - uncontrolled angina pectoris - congestive heart failure NYHA Grade 2 - ventricular arrhythmias requiring continuous therapy - supraventricular arrhythmias including AF, which are uncontrolled - Torsades de Pointes - haemorrhagic or thrombotic stroke, including transient ischaemic attacks or any - other central nervous system bleeding |
|  | Resting ECG with measurable QTc interval of >470ms msec at 2 or more time points within a 24 hour period. |
|  | Concomitant medications known to prolong QT interval, or with factors that increase the risk of QTc prolongation or risk of arrhythmic events (such as heart failure, hypokalaemia, congenital long QT syndrome, family history of long QT syndrome), or unexplained sudden death under 40 years of age. Inability to discontinue medication with agents designated as having a risk of Torsades de Pointes due to QT prolongation (see Appendix 5) |
|  | Left ventricular (LV) dysfunction (LVEF outside institutional range of normal) by MUGA or echocardiogram. |
|  | Current malignancies of other types, with the exception of adequately treated cone-biopsied in situ carcinoma of the cervix uteri and basal or squamous cell carcinoma of the skin. Cancer survivors, who have undergone potentially curative therapy for a prior malignancy who have no evidence of that disease currently are eligible for the trial. |
|  | Prior bone marrow transplant or have had extensive radiotherapy to greater than 25% of bone marrow within eight weeks of starting trial |
|  | Patients participating in or planning to participate in another interventional clinical trial whilst on this study. Participation in an observational trial is acceptable. |
|  | Any other condition which in the Investigator’s opinion would not make the patient a good candidate for the clinical trial. |

**Prohibited concomitant medications**

Potent and moderate inhibitors and inducers of CYP3A4/5 if taken within the stated

washout periods:

*Inhibitors (competitive):* ketoconazole, itraconazole, indinavir, saquinovir, nelfinavir, atazanavir, amprenavir, fosamprenavir, troleandomycin, telithromycin, fluconazole, nefazodone, cimetidine, aprepitant, miconazole, fluvoxamine (1 week minimum wash-out period), amiodarone (27 week minimum wash-out period)

*Inhibitors (time dependent):* erythromycin, clarithromycin, verapamil, ritonavir, diltiazem (2 week minimum wash-out period)

*Inducers*: phenytoin, rifampicin, St. John's Wort, carbamazepine, dexamethasone, primidone, griseofulvin, carbamazepine, barbiturates, troglitazone, pioglitazone, oxcarbazepine, nevirapine, efavirenz, rifabutin (3 week minimum wash-out period) and phenobarbitone (5 week minimum washout period)

Potent and moderate inhibitors and inducers of CYP2C8 if taken within the stated

washout periods:

*Inhibitors:* Gemfibrozil, trimethoprim, glitazones, montelukast, quercetin (1 week minimum wash-out period)

*Inducers:* Rifampicin (3 week minimum wash-out period)

**Methods**

**PK assay**

Outline

This method is applicable to the analysis of AZD2014 in human plasma treated with K2EDTA anticoagulant. AZD2014 and the internal standard (ISTD), 13C22H2AZ12729279 are extracted from human plasma by solid-phase extraction (SPE). After evaporation under nitrogen, the residue is reconstituted and analysed using liquid chromatography (LC) with tandem mass spectrometric detection (MS/MS). The standard curve range is from 20.0 to 20,000 ng/mL for AZD2014, using a plasma sample volume of 0.0250 mL.

Solid phase extraction

Condition the 96 Well Format SPE (Oasis HLB, 30 μm, 10 mg): Add 500 μL of methanol to the SPE cartridges and apply low pressure to let the solvent pass. Add 500 μL of 10 mM ammonium formate in water to the SPE cartridges and apply low pressure to let the solvent pass. Load the entire 500 μL of the samples onto the SPE cartridges and apply very low pressure to let samples pass. Wash the SPE cartridges with 500 μL of 10 mM ammonium formate in water [MA1] and apply low pressure to let the solvent pass. Wash the SPE cartridges with 500 μL of methanol:water:ammonium hydroxide (30:65:5, v:v:v) and apply low pressure to let the solvent pass. Dry the cartridges using maximum pressure for at least 20 seconds. Blot the tips dry on paper towelling. Place the cartridges on top of a clean pre-labelled Axygen 96-well plate. Add 300 μL of 2% formic acid in methanol to each SPE cartridge. Let the cartridges stand for 1 minute on top of the 96-well plate. Slowly elute the samples into the Axygen 96-well collection plate using minimal pressure. After complete elution increase pressure to remove any remaining drips and dry the SPE packing material. Evaporate the extracts to dryness under a stream of nitrogen using the SPE Dry-96. Set the upper and lower gas temperature to 40°C. Set both upper and lower gas flow to approximately 50 L/min. The total evaporation time is approximately 20 minutes. Using an Eppendorf repeating pipette or Tomtec, reconstitute the samples by adding 500 μL of 10mM ammonium formate:methanol (3:2, v:v) [RC1] to each sample. Cover the 96-well plate with a dimpled sealing mat and vortex-mix at a low speed for approximately 1 minute. Perform a 20x dilution of each sample using a Tomtec. Transfer 25 μL of each sample from Step 5 to a clean pre-labeled Axygen 96-well plate. Dilute each sample by adding 475 μL of 10mM ammonium formate:methanol (3:2, v:v) [RC1]. Cover the 96-well plate with a dimpled sealing mat and vortex-mix at a low speed for approximately 1 minute. Keep sample extracts at refrigerated conditions if needed prior to injection.

Chromatographic Conditions

Column: Waters, Acquity UPLC® BEH C18, 2.1 x 50 mm, 1.7 μm particle size

Pre-filter: Waters, Acquity column inline filter

Column Temp.: 60°C

Mobile Phase: A: 10 mM ammonium formate in water B: methanol

Gradient Program: Initial Conditions: 0.600 mL/min; 50 % B

Time

(minutes) Module Function Value (%)

0.30 Pumps Pump B Conc. 50

2.00 Pumps Pump B Conc. 75

2.10 Pumps Pump B Conc. 98

2.50 Pumps Pump B Conc. 98

2.60 Pumps Pump B Conc. 50

3.50 System Controller Stop

Flow Rate: 0.600 mL/min

Back Pressure: 410 Bar (Typical)

Sample Tray Temp: Refrigerated temperature (2 to 8°C)

Injection Volume: 5-10 μL (Typical); Not to exceed 20.0 μL

Rinse Port Injector

Wash Solution: 10mM ammonium formate:methanol (3:2, v:v)

Rinse Pump Injector

Wash Solution: Methanol: DMSO (4:1, v:v)

Needle Stroke: 47 mm

Rinse Pump Setting: Rinse pump → Rinse port

Rinse Volume: 500 μL

Rinse Mode: Before and After Sampling

Rinse Dip Time: 3 seconds

Rinse Time: 1 second

Acquisition Time: Approximately 3.5 minutes

Cycle Time: Approximately 4.0 minutes (injection start to next injection start)

Mass Spectrometer Parameters

Mass Spectrometer: Sciex API 4000

Ionization: Positive Ion Electrospray (ESI+)

Mode: MRM

IonSpray Voltage: 4500V

TurboIon Spray Temp: 600°C

Curtain Gas Type: Nitrogen Setting: 30

CAD Gas Type: Nitrogen Setting: 6

Nebulizing Gas (Gas1) Type: Nitrogen Setting: 50

Auxiliary Gas (Gas 2) Type: Nitrogen Setting: 70

Needle Position: Y = 5 mm

X = 5 mm

**Pharmacodynamic biomarker analysis: peripheral blood mononuclear cells (PBMCS) and platelet rich plasma (PRP)**

Peripheral blood mononuclear cells (PBMCs) fixed with 4% formaldehyde were incubated for ten minutes with 4% fetal calf serum (FCS), 0.1% Triton X-100, PBS as a blocking agent followed by staining with excess anti-phospho 4EBP1 (Thr37/46) (236B4) Rabbit monoclonal antibody Alexa 488 conjugate (2846, Cell Signaling Technology, Massachusetts, USA). Stained samples were washed once in 4% FCS, 0.1% Triton X-100, PBS and once in 0.1% Triton X-100, PBS. Stained samples were suspended in an appropriate volume of 0.1% Triton X-100, PBS and analysed using a FACSCantoII. A rabbit IgG isotype control (4340, Cell Signaling Technology, Massachusetts, USA) was used to set the negative population and assess the percentage of monocytes positive for phosphorylated 4EBP1. During validation the inter-assay precision, ranged from 2.8 to 3.3%.

pAkt was assessed in platelet rich plasma (PRP) using a sandwich immunoassay as per manufacturer’s instructions (K11100D, Meso Scale Discovery, Maryland, USA) which consisted of a multiplex assay with  plates pre-coated with phospho(Ser473) Akt and total Akt capture antibodies. These plates were blocked for 1 hour, incubated with sample for 1 hour, and finally incubated with an anti-total Akt detection antibody conjugated to MSD-SULFO-TAG for 1 hour before being detected on a Meso Scale Discovery Sector Imager 6000 using electrochemiluminescence in order to determine the percentage of Akt phosphorylated. During validation the inter-assay precision ranged from 6.75 to 7.17%.

Inter-assay precision for both methods was determined as the co-efficient of variance (CV) of 3 independent measurements across 3 days.

**Genomic Sequencing**

DNA was extracted from formalin-fixed and paraffin-embedded (FFPE) tumour blocks using the FFPE Tissue DNA kit (Qiagen). DNA was quantified with the Quant-iT high-sensitivity PicoGreen double-stranded DNA Assay Kit (Invitrogen). Sequencing libraries were constructed from 40 ng of DNA using a customized Generead DNAseq Mix-n-Match v2 panel (Qiagen) covering 4841 amplicons (310,077 bp) across 67 genes. Libraries were run using the MiSeq Sequencer (Illumina) at a mean of 650X (tissue) and 1018X (cfDNA). FASTQ files were generated using the Illumina MiSeq Reporter v2.5.1.3. Sequence alignment and mutation calling were performed using BWA tools and the GATK variant annotator by the Qiagen GeneRead Targeted Exon Enrichment Panel Data Analysis Web Portal.
