# Supplementary material for: Vistusertib (dual m-TORC1/2 inhibitor) in combination with paclitaxel in patients with high-grade serous ovarian and squamous non-small-cell lung cancer
Source: Ann Oncol. 2018 Jul 17;29(9):1918–25. doi: 10.1093/annonc/mdy245 (PMC6158767; doi:10.1093/annonc/mdy245)
Supplement: Supplementary Table 1 [file mdy245_table_1_revised.docx]

| **Characteristics** | **Total on Dose Escalation** |
| --- | --- |
|  | ***N* (%)** |
| **Total number of patients** | 22 |
| **Primary tumour** |  |
| Ovary | 8 (36) |
| Lung adenocarcinoma | 4 (18) |
| Lung squamous | 2 (9) |
| Breast | 1 (5) |
| Endometrial | 4 (18) |
| Gastroesophageal | 1 (5) |
| Cervix | 2 (9) |

**Supplementary Table 1. Demographic profile of patients treated in the dose escalation cohort**
