# Supplementary material for: Vistusertib (dual m-TORC1/2 inhibitor) in combination with paclitaxel in patients with high-grade serous ovarian and squamous non-small-cell lung cancer
Source: Ann Oncol. 2018 Jul 17;29(9):1918–25. doi: 10.1093/annonc/mdy245 (PMC6158767; doi:10.1093/annonc/mdy245)
Supplement: Supplementary Table 2 [file mdy245_table_2_revised.docx]

| **Characteristics** | **Subtypes** | **OVARIAN Expansion (50 mg AZD2014/80 mg Paclitaxel)** | **LUNG Expansion (25 mg AZD2014/80 mg Paclitaxel)** |
| --- | --- | --- | --- |
|  |  | ***N* (%)** | ***N* (%)** |
| Primary tumour | High grade serous ovarian | 27 (100) | - |
|  | Lung squamous | - | 24 (100) |
| Gender | Male | - | 11 (46) |
|  | Female | 27 (100) | 13 (54) |
| Age (years) | 18 - 64 | 11 (41) | 11 (44) |
|  | ≥65 | 16 (59) | 13 (56) |
| ECOG performance status | 0 | 7 (26) | 4 (17) |
|  | 1 | 20 (74) | 20 (83) |
| Median previous lines of therapy |  | 3 (range: 1 to 12) | 2 (range: 1 to 3) |
| Previously treated with taxane (incl. docetaxel or paclitaxel) |  | 26 (96) (Previous weekly taxol = 3) | 6 (25) |
| RECIST response |  | 13 PR | 8 PR (33) |
| Median number of weeks on treatment |  | 21 | 18 |
| Ongoing on 1-Oct-2017 |  | 1 | 1 |
| Platinum sensitivity | Platinum refractory | 6 (22) | 1 (4) |
|  | Platinum resistance (< 6 months) | 11 (41) | 15 (63) |
|  | Platinum intermediate sensitive (6 - 12 months) | 9 (33) | 4 (17) |
|  | Platinum sensitive (> 12 months) | 1 (4) | 4 (17) |

**Supplementary Table 2. Demographic profile of patients treated in the dose expansion cohort**
