# Supplementary material for: Vistusertib (dual m-TORC1/2 inhibitor) in combination with paclitaxel in patients with high-grade serous ovarian and squamous non-small-cell lung cancer
Source: Ann Oncol. 2018 Jul 17;29(9):1918–25. doi: 10.1093/annonc/mdy245 (PMC6158767; doi:10.1093/annonc/mdy245)
Supplement: Supplementary Table 3A 3B [file mdy245_table_3a_3b.docx]

| **Lung Expansion 50 mg Vistusertib (*N*=17)** | | | | |
| --- | --- | --- | --- | --- |
| **Adverse Event, *N* (%)** | **Gr.1 - Gr.2** | **Gr.3 - Gr.4** | **Total** | **%** |
| Fatigue | 6 | 4 | 10 | 59 |
| Skin rash | 8 | 1 | 9 | 53 |
| Anorexia/Appetite loss/Weight loss | 8 | 0 | 8 | 47 |
| Diarrhoea | 5 | 2 | 7 | 41 |
| Neuropathy | 6 | 0 | 6 | 35 |
| Thrush (oral) | 6 | 0 | 6 | 35 |
| Anaemia | 4 | 1 | 5 | 29 |
| Bronchitis/Bronchial or respiratory infection | 4 | 1 | 5 | 29 |
| Dyspnea/Shortness of breath | 5 | 0 | 5 | 29 |
| Mucositis/Oral mucositis/Mouth ulcers | 5 | 0 | 5 | 29 |
| Nausea | 4 | 1 | 5 | 29 |
| Vomiting | 5 | 0 | 5 | 29 |
| Pneumonitis/Pneumonia | 3 | 1 | 4 | 24 |
| Cough | 3 | 0 | 3 | 18 |
| Dry skin/Eczema | 3 | 0 | 3 | 18 |
| Dyspepsia/Indigestion/GI tox | 3 | 0 | 3 | 18 |
| Hyperglycaemia | 2 | 1 | 3 | 18 |
| Leucopenia | 2 | 1 | 3 | 18 |
| Neutropenia | 2 | 1 | 3 | 18 |
| Aches and pain | 2 | 0 | 2 | 12 |
| Alopecia | 2 | 0 | 2 | 12 |
| Dry mouth | 2 | 0 | 2 | 12 |
| Dysgeusia | 2 | 0 | 2 | 12 |
| Elevated ALT | 2 | 0 | 2 | 12 |
| Elevated AST | 2 | 0 | 2 | 12 |
| Malaise/Sweats | 2 | 0 | 2 | 12 |
| Paronychia/Nail changes | 2 | 0 | 2 | 12 |
| Sore throat | 2 | 0 | 2 | 12 |
| Cellulitis | 0 | 1 | 1 | 6 |
| Drug reaction | 1 | 0 | 1 | 6 |
| Epistaxis | 1 | 0 | 1 | 6 |
| Glucose intolerance | 1 | 0 | 1 | 6 |
| Headache | 1 | 0 | 1 | 6 |
| Hyperbilirubinaemia | 1 | 0 | 1 | 6 |
| Myalgia | 1 | 0 | 1 | 6 |
| Red skin (face) | 1 | 0 | 1 | 6 |
| Trigeminal nerve disorder | 1 | 0 | 1 | 6 |
| UTI | 0 | 1 | 1 | 6 |
| **Total** | **110** | **16** | **126** |  |

**Supplementary Table 3A. Toxicity profile of patients treated on the squamous non-small cell lung cancer expansion cohort**

Patients administered with 80 mg/m^2^ paclitaxel with 50 mg vistusertib bd 3/7 days for 6/7 weeks, dosing starting concurrently on day 1 of each week

| **Lung Expansion 25 mg Vistusertib (*N*=24)** | | | | |
| --- | --- | --- | --- | --- |
| **Adverse Event, *N* (%)** | **Gr.1 - Gr.2** | **Gr.3 - Gr.4** | **Total** | **%** |
| Anaemia | 14 | 0 | 14 | 58 |
| Fatigue | 13 | 1 | 14 | 58 |
| Alopecia | 13 | 0 | 13 | 54 |
| Neuropathy | 11 | 0 | 11 | 46 |
| Nausea | 10 | 0 | 10 | 42 |
| Bronchitis/Bronchial or Respiratory infection | 4 | 4 | 8 | 33 |
| Diarrhoea | 8 | 0 | 8 | 33 |
| Skin rash | 7 | 0 | 7 | 29 |
| Dyspepsia/Indigestion | 5 | 0 | 5 | 21 |
| Loss of appetite/Anorexia/Weight loss | 4 | 1 | 5 | 21 |
| Mucositis/Mouth ulcers/Stomatitis | 5 | 0 | 5 | 21 |
| Arthalgia/Myalgia | 4 | 0 | 4 | 17 |
| Leucopenia | 4 | 0 | 4 | 17 |
| Dry skin/Eczema | 3 | 0 | 3 | 13 |
| Dyspnoea | 3 | 0 | 3 | 13 |
| Lesion/Lump on skin | 3 | 0 | 3 | 13 |
| Nail loss/discoloration | 3 | 0 | 3 | 13 |
| Pneumonitis/Pneumonia | 3 | 0 | 3 | 13 |
| Vomiting | 3 | 0 | 3 | 13 |
| Abdominal cramps/pain | 2 | 0 | 2 | 8 |
| Cough | 2 | 0 | 2 | 8 |
| Elevated creatinine | 2 | 0 | 2 | 8 |
| Epistaxis | 2 | 0 | 2 | 8 |
| Hyperkalaemia | 2 | 0 | 2 | 8 |
| Hypophosphataemia | 1 | 1 | 2 | 8 |
| Leg oedema | 1 | 1 | 2 | 8 |
| Neutropenia | 1 | 1 | 2 | 8 |
| Tachycardia | 2 | 0 | 2 | 8 |
| Coryzal symptoms | 1 | 0 | 1 | 4 |
| Dehydration | 0 | 1 | 1 | 4 |
| Dry mouth | 1 | 0 | 1 | 4 |
| Early satiety | 1 | 0 | 1 | 4 |
| Haemoptysis | 1 | 0 | 1 | 4 |
| High magnesium | 1 | 0 | 1 | 4 |
| High urea | 1 | 0 | 1 | 4 |
| Hyponatraemia | 0 | 1 | 1 | 4 |
| Hypotensive | 0 | 1 | 1 | 4 |
| Increased glucose | 1 | 0 | 1 | 4 |
| Long nails | 1 | 0 | 1 | 4 |
| Low magnesium | 1 | 0 | 1 | 4 |
| Naval discoloration | 1 | 0 | 1 | 4 |
| Pruritis | 1 | 0 | 1 | 4 |
| Thrush | 1 | 0 | 1 | 4 |
| Wound breakdown | 1 | 0 | 1 | 4 |
| Total | 148 | 12 | 161 |  |

**Supplementary Table 3B.** **Toxicity profile of patients treated on the squamous non-small cell lung cancer expansion cohort**

Patients administered with 80 mg/m^2^ paclitaxel with 25 mg vistusertib bd 3/7 days for 6/7 weeks, dosing starting concurrently on day 1 of each week treated at paclitaxel 80 mg/m^2^/week and vistusertib 25 mg bd 3/7.
